# Relationships of the gut microbiome with cognitive development among healthy school-age children

**DOI:** 10.3389/fped.2023.1198792

**Published:** 2023-05-19

**Authors:** Yelena Lapidot, Maayan Maya, Leah Reshef, Dani Cohen, Asher Ornoy, Uri Gophna, Khitam Muhsen

**Affiliations:** ^1^ Department of Epidemiology and Preventive Medicine, School of Public Health, the Sackler Faculty of Medicine, Tel Aviv University, Tel Aviv, Israel; ^2^ The Shmunis School of Biomedicine and Cancer Research, Faculty of Life Sciences, Tel Aviv University, Tel Aviv, Israel; ^3^ Adelson School of Medicine, Ariel University, Ariel, Israel; ^4^ Department of Medical Neurobiology, The Hebrew University Hadassah Medical School, Jerusalem, Israel

**Keywords:** gut microbiome, children, healthy, school age, cognitive development, socioeconomic status

## Abstract

**Background:**

The gut microbiome might play a role in neurodevelopment, however, evidence remains elusive. We aimed to examine the relationship between the intestinal microbiome and cognitive development of school-age children.

**Methods:**

This cross-sectional study included healthy Israeli Arab children from different socioeconomic status (SES). The microbiome was characterized in fecal samples by implementing 16S rRNA gene sequencing. Cognitive function was measured using Stanford-Binet test, yielding full-scale Intelligence Quotient (FSIQ) score. Sociodemographics and anthropometric and hemoglobin measurements were obtained. Multivariate models were implemented to assess adjusted associations between the gut microbiome and FSIQ score, while controlling for age, sex, SES, physical growth, and hemoglobin levels.

**Results:**

Overall, 165 children (41.2% females) aged 6–9 years were enrolled. SES score was strongly related to both FSIQ score and the gut microbiome. Measures of α-diversity were significantly associated with FSIQ score, demonstrating a more diverse, even, and rich microbiome with increased FSIQ score. Significant differences in fecal bacterial composition were found; FSIQ score explained the highest variance in bacterial β-diversity, followed by SES score. Several taxonomic differences were significantly associated with FSIQ score, including *Prevotella*, *Dialister*, *Sutterella*, *Ruminococcus callidus,* and *Bacteroides uniformis*.

**Conclusions:**

We demonstrated significant independent associations between the gut microbiome and cognitive development in school-age children.

## 1. Introduction

The gut microbiome is important in health and disease (1, 2). The microbiota is increasingly recognized for its ability to influence the nervous system and several complex behaviors of the host, by modulation of neurodevelopment through the microbiome-gut-brain axis (3, 4).

The gut-brain axis is characterized by a bidirectional communication between the gut and the brain, that might modify both gastrointestinal and nervous systems function, influencing emotion and cognition (4). Preclinical and clinical studies showed that variations in microbiota composition contribute to various cognitive states, including functional brain connectivity, depression, stress, anxiety, and autism spectrum disorder (5–12). The mechanisms underlying these relationships are not fully understood, but a compelling hypothesis is that gut microbiota variation during childhood with vulnerable neurodevelopmental window, might influence both mental and cognitive outcomes (13).

The first years of life are characterized by intense structural and functional changes in the brain and thus are critical for neurodevelopmental plasticity (14). Intriguingly, these changes occur simultaneously with dynamic intestinal microbiome alterations, thus raising the possibility of dialogue between the microbes that inhabit the gastrointestinal tract and the brain in early life (15, 16). Animal models demonstrated that early life gut microbiome influences later neurodevelopment (17). However, evidence from human populations remains limited and focused merely on infancy, demonstrating the association between the intestinal microbiome with both temperament (18, 19) and cognitive performance (12, 20). Evidence suggests substantial functional and taxonomic differences in the gut microbiota of healthy children compared to those of adults, suggesting that the development of gut microbiome may continue into school age and more slowly than previously thought and that the gut microbiota of children may be more malleable to environmental factors than that of adults (21, 22). Moreover, neurodevelopment remains an ongoing critical process during the early to middle childhood years (23), nevertheless the association between the microbiome and cognitive development during school age remains elusive.

Environmental exposures, including socioeconomic status (SES) play a critical role in both intestinal microbiome (24, 25) and neurologic development (26, 27). Moreover, iron deficiency anemia, a main risk factor for diminished neurodevelopmental and cognitive abilities in children (28–30), was linked with the gut microbiome, in both animal models and human studies (31–33). However, the interconnection between the microbiome, environmental exposures, and cognitive function in childhood is not fully clear. To address these gaps, we examined the association between the intestinal microbiome and cognitive development of school-age children, with the possible intermediating effect of environmental exposures, including sociodemographics, physical growth, and nutritional status. Our working hypothesis was that the gut microbiome might be related to cognitive development of healthy school-age children, independent of potential confounders.

## 2. Materials and methods

### 2.1. Study population and design

This cross-sectional study focused on a population under transition, the Israeli Arab population, the main ethnic minority in Israel. This population comprises 20% of the Israeli population (34, 35), while 75% are Jews and 5% belong to other population groups. The Arab population has lower educational levels and SES compared to the Jewish population (35, 36), but there is an ongoing improvement in the educational level and health indicators among Arabs. Access to care is universal in Israel, due to the universal health insurance law (37).

This study was conducted in 2007–2009, in three Arab villages in Hadera sub-district. In 2007, there were about 153,000 Muslim Arab residents living in this region, with 3,921 live births (34). One village had approximately 14,000 residents during the study period and the other two villages had about 10,000 residents each. According to the Central Bureau of Statistics, one village belonged to cluster 2 SES, one belonged to cluster 3 SES, and the third village belonged to cluster 4 SES. The clusters are on a scale of 1–10, the lower the index, the lower the SES (36). At the national level, these villages are of low and intermediate SES levels (36). Given the SES differences across the villages, they were referred herein as village A = high SES, village B = intermediate SES, and village C = low SES. The drinking water supply in these villages is piped, and all households are connected to the national electricity company similar to the rest of the country. Connection to the internet and cable television is also available.

In this cross-sectional study, we examined the gut microbiome in archived stool samples obtained from healthy children who participated in a study on gastrointestinal tract infections. Briefly, in 2003–2004, a cohort of 289 healthy children aged 3–5 years from three villages of different SES were recruited. In 2007–2009, a follow-up was performed among 196 children at age 6–9 years (38, 39). Overall, 176 children who provided stool samples had sufficient material for 16S rRNA sequencing. Eight children were excluded due to medical conditions that might affect cognitive function directly (thalassemia minor, type-1 diabetes, Glucose-6-phosphate dehydrogenase deficiency with anemia, major heart defect, panhypopituitarism, hemophilia, and significant developmental delay). Three additional children with missing IQ scores were omitted from the analysis, thus leaving 165 participants in the analysis.

### 2.2. Data collection

Information on household and socioeconomic characteristics was obtained via personal interviews with the mothers, by trained Arabic-speakers interviewers. The questionnaire included information on age, sex, the village of residence, maternal education, maternal age, paternal education, monthly family income, number of persons living in the household, and number of rooms in the household. Crowding index was calculated by dividing the number of people living in a household by the number of rooms in a household. Data were collected on early life determinants e.g., birth weight, breastfeeding, and daycare center attendance.

### 2.3. Current hemoglobin levels

Blood collected by finger lancing was used for hemoglobin measurement using a portable hemoglobinometer (Hemocue Hb 201+, Sweden). Hemoglobin was assessed as an indicator of nutritional status.

### 2.4. Anthropometric measurements

Anthropometric measurements were performed by trained registered nurses. Body weight was measured to the nearest 0.1 kilogram using an analog scale (calibrated before use), and height (to the nearest 0.1 centimeter) with a stadiometer. Information on anthropometric measurements in early childhood (ages 18–30 months) was obtained from medical records. *Z* scores of height for age (HAZ), weight for height (WAZ), and body mass index for age (BMIZ) were calculated using Epi/Info software [Center for Disease Control and Prevention, Atlanta, Georgia (CDC)] based on the 2,000 CDC growth reference curves. BMI was calculated as weight (kg)/height (m)^2^.

### 2.5 . Socioeconomic status (SES)

Multiple SES indicators were examined: (1) community SES rank as classified by the Israel Central Bureau of Statistics, (2) household socioeconomic characteristics: (a) maternal education, (b) paternal education, (c) crowding index, and (d) reported family income. We used these variables to generate a composite SES score, based on confirmatory factor analysis. The analysis was implemented using “Principal Axis” method, including rotation with “varimax” (*r* package psych). Since maternal education was significantly correlated with parental education (Pearson's *r* = 0.46), we included only maternal education level in the analysis. The selected variables were tested with Bartlett's test of homogeneity of variances (*p*-value <0.0001) and Kaiser–Meyer–Olkin factor adequacy resulting in adequate scores for all selected variables: village of residence = 0.7, crowding index = 0.7, maternal education = 0.7, reported family income = 0.68. The newly generated SES score was composed of a combination of the standardized loadings, based on the correlation matrix of the selected variables (Supplementary Figure S1).

### 2.6. Assessment of cognitive function

Cognitive function was measured by Intelligence Quotient (IQ) score using Stanford-Binet-5th edition (*SB5*) test, performed by a trained Arabic speaking psychologist (39). The following parameters were assessed: full-scale IQ (FSIQ), non-verbal IQ and verbal IQ. The test was performed at standard conditions, lasting 45–60 min. The *SB5* was scored with the *SB5 Scoring Pro*, a Windows®-based software program. Since FSIQ is highly correlated with non-verbal and verbal IQ (Pearson's correlation *r* = 0.95 and *r* = 0.94 respectively), FSIQ score was selected as the main outcome variable in this study. The psychologist was masked to background information of the participants.

### 2.7. Samples collection, DNA extraction and bacterial DNA amplification

Fresh stool samples were obtained from the children using collection plastic cups and transferred on ice to the laboratory at Tel Aviv University. Samples were divided stored at −80°C until testing. All samples underwent a single thaw prior to DNA extraction. DNA was extracted from 180 to 220 mg of fecal material from each sample using the QIAamp® Fast DNA Stool Mini Kit (Qiagen, Valencia, CA) following the manufacturer's instructions (40) and stored at −20°C until shipment to the Sequencing Core at the University of Illinois. Genomic DNA was prepared for sequencing using a two-stage amplicon sequencing workflow (41). Initially, genomic DNA was amplified via PCR using primers targeting the V4 region of microbial 16S ribosomal RNA (rRNA) genes. The primers, 515F modified and 806R modified (42), contained 5' linker sequences compatible with Access Array primers for Illumina sequencers (Fluidigm, South San Francisco, CA). The PCR assays were performed in a total volume of 10 µl using MyTaq™ HS 2X Mix (Bioline) with primer concentrations at 500 nM. Thermocycling conditions were as follows: 95°C for 5 min (initial denaturation), followed by 28 cycles of 95°C for 30 s, 55°C for 45 s, and 72°C for 30 s. One microliter of the PCR product from each reaction was transferred to the second-stage PCR assay. Each second-stage reaction was conducted in a final volume of 10 µl using MyTaq HS 2X mix, and each well contained a unique pair of Access Array primers containing Illumina sequencing adapters, single index sample-specific barcode, and linker sequences. Thermocycling conditions were as follows: 95°C for 5 min (initial denaturation), followed by 8 cycles of 95°C for 30 s, 60°C for 30 s, and 72°C for 30 s. Libraries were pooled and purified using 0.6× concentration of AMPure XP beads to remove short fragments below 300 bp. Pooled libraries were loaded onto a MiniSeq sequencer (Illumina, San Diego, CA) with 15% phiX spike-in and paired-end 2 × 153 base sequencing reads.

### 2.8. Statistical analyses

Quality control analysis of demultiplexed sequences was performed using the Deblur (43) workflow, following the construction of a phylogenetic tree (mafft-fasttree) and taxonomy assignment with QIIME2 (44). The quality process with Deblur uses sequence error profiles to obtain putative error-free sequences, referred to as “sub” operational taxonomic units (s-OTU). Taxonomic composition was assigned to the s-OTUs using a pre-trained Naive Bayes classifier, trained on the Greengenes (45) 13_8 99% OTUs. Downstream analysis was conducted using R version 4.0.3. Diversity analysis was calculated at rarefaction depth of 11,158. Bacterial α-diversity, which quantifies the intra-sample diversity, i.e., the distribution of species abundances in a given sample, was estimated using Shannon's diversity and Pielou's evenness indexes (46) and compared across independent variables using multivariate analysis of variance (ANOVA) tests. β-diversity, which measures dis-similarities between samples (46), was calculated using the Bray-Curtis dissimilarity index, the Jensen-Shannon divergence (JSD), and the phylogenetic weighted and unweighted Unifrac distances. Permutational multivariate analysis of variance (PERMANOVA) was used to test differences in overall microbiome composition [vegan; Adonis (47)], implementing a multivariate model with the covariates: age, sex, SES score, hemoglobin levels, HAZ at age 18–30 months and current BMIZ scores. The Analysis of Composition of Microbiomes (48) (ANCOM) was applied for the identification of differentially abundant features in association with FSIQ scores, with the false discovery rate (FDR) level set to 0.05. ANCOM uses a linear framework to statistically detect features whose composition varies across FSIQ scores, while controlling for other covariates of interest (a linear model comprised of the abovementioned covariates). A feature was considered significantly varying in composition across an independent variable of interest at a detection level of ≥0.6, meaning that the feature composition varied across the independent variable with respect to 60% of reference features. Non-parametric Spearman's correlation coefficient was used to evaluate the association between α-diversity indices and FSIQ scores.

Differences in demographic characteristics across the study villages were examined using one-way ANOVA for continuous variables, the Kruskal–Wallis *H* test for rank-based variables and the *χ*^2^ test for categorical variables. Post-hoc pairwise comparisons were conducted using Games–Howell test, including multiple comparisons correction with FDR.

### 2.9. Ethical Approval

The Institution Review Board of Hillel Yaffe Medical Center (approval number 6/2005, year of approval 2005) and the Ethics Committee of Tel Aviv University approved the study (approval year 2018). Written informed consent was obtained from the parents of the participants.

## 3. Results

### 3.1. Demographic characteristics of the study participants

Data from 165 children (41.2% females) who provided stool specimens and underwent a cognitive assessment were included in the analysis. The participants’ mean age was 7.8 years, [SD = 0.9], with significant differences between the villages (*p* = 0.019). The composite SES score ranged from −2.1 to 4.6 [mean 2.1 (SD = 1.4); Supplementary Figure S1B] and was profoundly different between the villages (*p* < 0.0001; Supplementary Figure S2A). Children from village C (low SES) had significantly worse SES indicators than children from villages A/B, but there were no significant differences between the villages in early life determinants, e.g., birth weight, breastfeeding, and attending a daycare. HAZ scores in infancy were significantly lower (*p* = 0.026) in children from village C (low SES) than children from villages A/B (high/intermediate SES). The mean BMIZ score at school age was higher among children from village C compared to villages A/B (*p* < 0.001) (Table 1). The mean FSIQ of this cohort was 98.8 [SD = 13.1] points. FSIQ score was lower among children from village C compared to villages A/B (Supplementary Figure S2B).

**Table 1:** Characteristics of the participants by the village of residence (*N* = 165).

	Villages A/B (intermediate/high SES)	Village C (low SES)	*p* value
Number of participants, (%)	100 (60.6%)	65 (39.4%)	–
Age, years, mean (SD)	7.9 (0.9)	7.6 (0.8)	0.019
Sex, females, *N* (%)	40 (40.0%)	28 (43.1%)	0.818
Household crowding index^a^, mean (SD)	1.4 (0.6)	2.6 (1.3)	<0.001
Household monthly income^b^			<0.001
Above average	16 (16.0%)	3 (4.6%)	
Average	34 (34.0%)	8 (12.3%)	
Below average	50 (50.0%)	54 (83.1%)	
Father education, years, mean (SD)	11.3 (3.4)	8.3 (3.5)	<0.001
Maternal education, years, mean (SD)	11.2 (3.5)	6.5 (3.7)	<0.001
SES score^c^, mean (SD)	3.0 (0.9)	0.8 (1.0)	<0.001
Birth weight (kg), mean (SD)	3.2 (0.5)	3.4 (0.5)	0.225
Breastfeeding, yes, *N* (%)	98 (98.0%)	57 (87.7%)	0.09
Age of introducing solid foods, months, mean (SD)	6.0 (2.9)	5.9 (2.6)	0.741
Daycare center attendance in early life, *N* (%)	20 (20.0%)	12 (18.8%)	0.697
Current hemoglobin level (g/dl), mean (SD)	12.6 (0.9)	12.5 (1)	0.567
Height for age *z*-score (age 18–30 months), mean (SD)	0.10 (0.80)	−0.20 (0.80)	0.026
Weight for age *z*-score, (age 18–30 months), mean (SD)	0.00 (0.9)	0.20 (1.0)	0.275
BMIZ score^c^, mean (SD)	0.20 (1.0)	0.82 (0.9)	<0.001
Full scale IQ score	105.0 (9.2)	89.2 (12.3)	<0.001

BMIZ, body mass index *z* score; SD, standard deviation; SES, socioeconomic status.

^a^ Household crowding: Number of people living in the household/Number of rooms in the household.

^b^ Household income: Household income as compared to the national average.

^c^ Individual level socioeconomic status score—a composite score based on confirmatory factor analysis including village of residence, maternal education, household crowding, and household income.

A significant positive association was found between the composite SES score and FSIQ scores (*p* < 0.0001 by ANOVA; Supplementary Figure S3A and Spearman's *r* = 0.61, *p* < 0.0001; Supplementary Figure S3B). FSIQ score was not correlated with hemoglobin level, nor with WAZ score at age 18–30 months (Supplementary Figures S4A,B). Significant associations were found between HAZ at age 18–30 months with FSIQ (Spearman's *r* = 0.22, *p* = 0.004) and between current BMIZ scores and with FSIQ (Spearman's *r* = −0.17, *p* = 0.025), Supplementary Figures S4C,D).

Based on these results and existing knowledge regarding the environmental effects of SES on both the microbiome (2, 49–51) and FSIQ (16, 52, 53), we examined the association between FSIQ score and fecal microbiome alterations, while adjusting for covariates that were associated with microbial alterations and FSIQ score.

### 3.2. The association between FSIQ score and bacterial α-diversity

**Figure 1:**
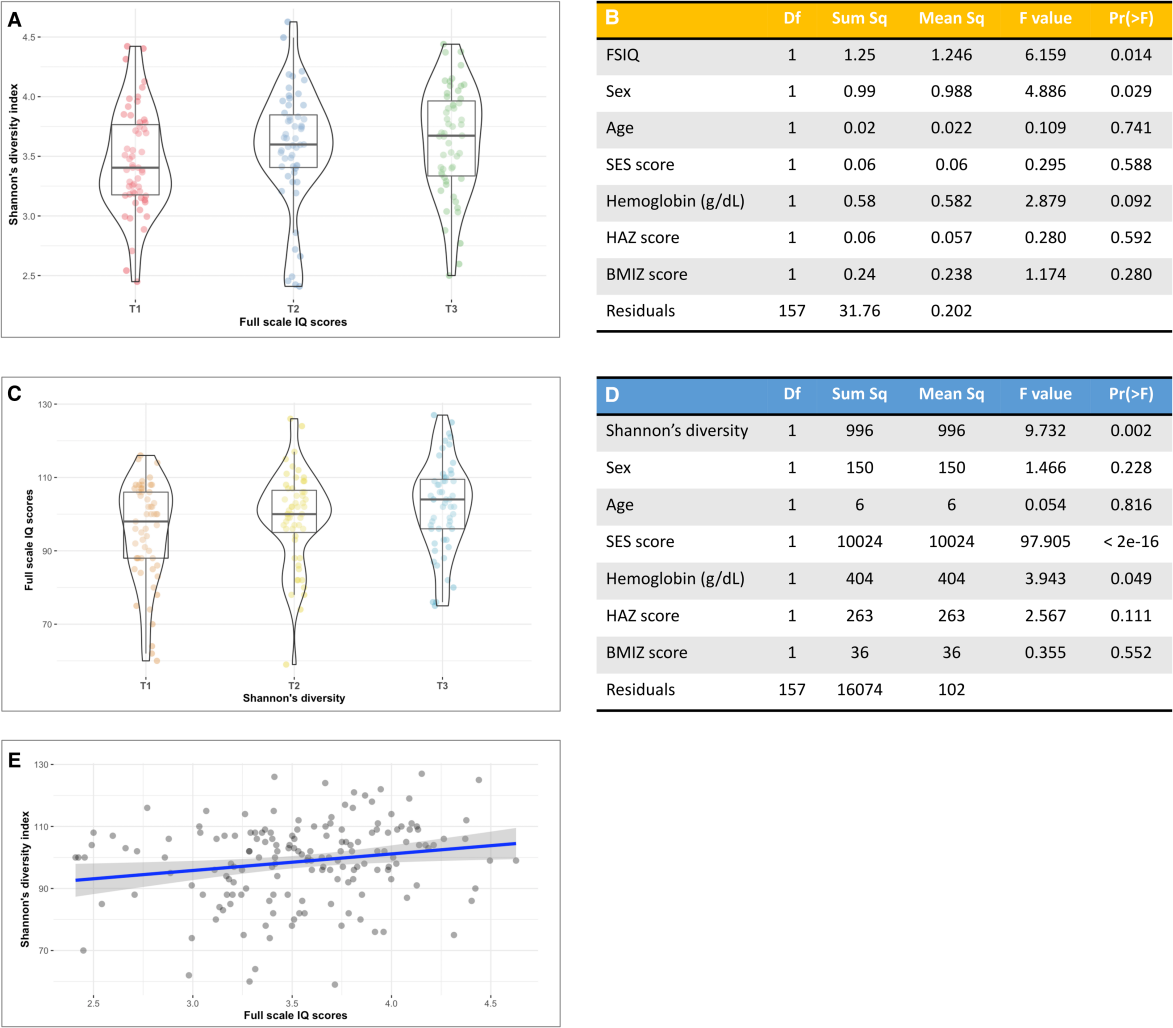
The association between full-scale IQ scores and bacterial α-diversity. (**A**) Box-violin plots of microbial diversity, measured by Shannon's diversity index, across tertiles of FSIQ scores, showing a significant increase in microbial α-diversity with increased FSIQ (*p* = 0.014). (**B**) Results of a multivariate analysis of variance displaying the association between FSIQ score and covariates of interest with bacterial diversity. FSIQ score and sex were significantly associated Shannon's bacterial α-diversity index (*F* = 6.16, *p* = 0.014 and *F* = 4.89 *p* = 0.029, respectively). (**C**) Box-violin plots of FSIQ scores across tertiles of Shannon's diversity index, show a significant increase in FSIQ scores with increased bacterial diversity (*p* = 0.002). (**D**) Results of a multivariate analysis of variance displaying the association between the individuals’ gut Shannon's diversity and covariates of interest with FSIQ scores. Bacterial α-diversity and SES scores were strongly associated with FSIQ score (*F* = 9.73, *p* = 0.014 and *F* = 97.91, *p* = 0.029, respectively), while hemoglobin levels had a more delicate albeit significant association (*F* = 3.94, *p* = 0.049). (**E**) The correlation between Shannon's α-diversity index and FSIQ score; Pearson's *r* = 0.20, *p* = 0.015. SES, socioeconomic status; FSIQ, full-scale IQ; HAZ, height for age *z*-score at age 18–30 months; BMIZ, body mass index *z*-score at age 6–9 years. *The *x* axis in figures (**A,C**) represents tertiles, T1 = lowest tertile, and T3 = highest tertile. **The mid line in the box plots [figures (**A**,**C**)] represents the median, the lower bound of the box represents the 25th percentile, the upper bound of the box represents the 75th percentile, the lowest point of the lower whisker represents the minimum and the highest point of the upper whisker represents the maximum. The violin plot implements a rotated kernel density plot on each side, adding information regarding the full distribution of the measured data; the width of the violin indicates the frequency.

Bacterial α-diversity as estimated by the Shannon's diversity index followed a normal distribution (Supplementary Figure S5). We found a significant positive association between Shannon's diversity and FSIQ score (Figure 1A). A multivariate analysis of variance model that adjusted for sex, age, SES score, hemoglobin level, HAZ at age 18–30 months and current BMIZ scores on bacterial diversity (Figure 1B), showed that FSIQ and sex were significantly associated with Shannon's diversity index (*F* = 6.16, *p* = 0.014 and *F* = 4.89 *p* = 0.029, respectively). A multivariate analysis that included FSIQ score as the dependent variable, showed a strong positive association between fecal α-diversity and FSIQ (*F* = 9.73, *p* = 0.002; Figure 1C). In this model, SES scores had the strongest association with FSIQ score (*F* = 97.91, *p* < 0.0001). Hemoglobin level was significantly associated with FSIQ score (*F* = 3.94, *p* = 0.049; Figure 1D). Bacterial α-diversity and FSIQ score were positively linearly correlated (Person's *r* = 0.20, *p* = 0.015; Figure 1E).

These associations were further strengthened by an estimation of bacterial α-diversity with Pielou's evenness index. There was a strong association between FSIQ score and increased species evenness (Supplementary Figure S6A), with FSIQ being the strongest and the only significant variable associated with altered species evenness (*F* = 11.32, *p* < 0.0001; Supplementary Table S1A). This model showed a borderline significant association between hemoglobin level and Pielou's evenness index (*F* = 2.91, *p* = 0.089). A multivariate model showed a significant relationship between FSIQ score and species evenness (*F* = 17.41, *p* < 0.0001), but the effect of SES (*F* = 91.13, *p* < 0.0001) on FSIQ score was stronger (Supplementary Table S1B). Significant positive linear correlation between FSIQ score and species evenness (Spearman's *r* = 0.24, *p* = 0.002; Supplementary Figure S6C).

### 3.3. The association of gut microbiome composition and FSIQ score

**Figure 2:**
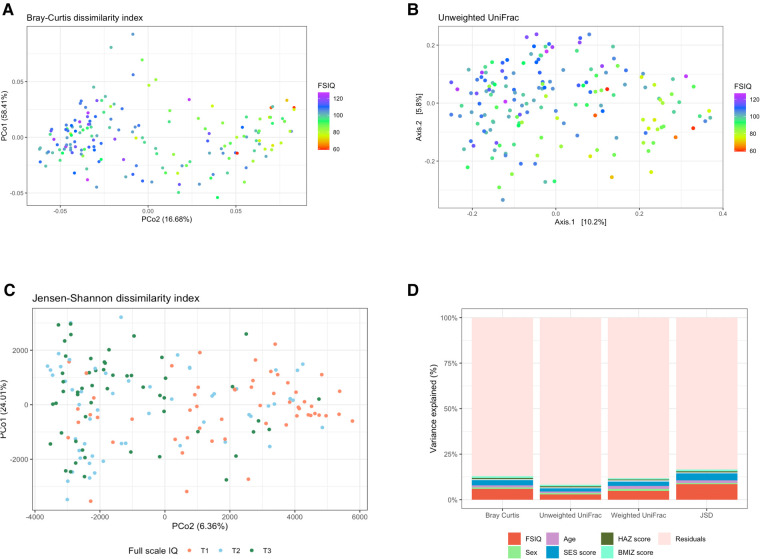
The association between full-scale IQ scores and bacterial β-diversity. (**A**) Principal coordinate analysis (PCoA) of the Bray-Curtis dissimilarity index, notably altered with changing FSIQ scores (*F* = 10.79, *p* = 0.001). (**B**) PCoA of the phylogenetic unweighted uniFrac distance matrix, significantly separated with altered FSIQ score (*F* = 5.04, *p* = 0.001). (**C**) PCoA of the Jensen-Shannon divergence (JSD), clearly separated according to FSIQ tertiles (*F* = 15.90, *p* = 0.001). (**D**) Stacked (100%) bar-plots of the explained variance in microbial beta diversity by the multivariate models. The FSIQ score explained most of the variance in all β-diversity measurements, followed by SES score. The JSD method explained the highest percentage of variance in microbial β-diversity (16.6%). FSIQ, full-scale IQ; PCoA, principal coordinate analysis; JSD, Jensen-Shannon divergence. *FSIQ scores in Figures (**C,D**) are represented as tertiles, T1 being the lowest tertile (FSIQ scores between 59 and 96), T2 the middle tertile (FSIQ scores between 97 and105), and T3 the highest tertile (FSIQ scores between 106 and 127). **The midline in the box plots [figure (**D**)] represents the median, the lower bound of the box represents the 25th percentile, the upper bound of the box represents the 75th percentile, the lowest point of the lower whisker represents the minimum and the highest point of the upper whisker represents the maximum. The violin plot implements a rotated kernel density plot on each side, adding information regarding the full distribution of the measured data; the width of the violin indicates the frequency.

We found significant differences in fecal bacterial composition, as measured by the Bray–Curtis dissimilarity index (*F* = 10.79, *R*^2 ^= 0.06, *p* = 0.001; Figure 2A, Supplementary Table S2A), the phylogenetic unweighted and weighted UniFrac distance matrixes (*F* = 5.04, *R*^2 ^= 0.03, *p* = 0.001, and *F* = 8.59, *R*^2 ^= 0.05, *p* = 0.001 relatively; Figure 2B, Supplementary Tables S2B, S2C), and by the JSD (*F* = 7.40, *R*^2 ^= 0.04, *p* = 0.001; Figure 2C, Supplementary Table S2D). All multivariate models included the covariates age, sex, SES score, hemoglobin, HAZ and BMIZ scores.

FSIQ score explained the highest variance in bacterial β-diversity as measured by the Bray-Curtis dissimilarity index, followed by the SES score (*F* = 10.79, *R*^2 ^= 0.06, *p* = 0.001, and *F* = 5.17, *R*^2 ^= 0.03, *p* = 0.001, respectively), the phylogenetic unweighted UniFrac distance matrix (*F* = 5.04, *R*^2^ = 0.029, *p* = 0.001, and *F* = 3.33, *R*^2 ^= 0.019, *p* = 0.001, respectively), the weighted UniFrac distance matrix (*F* = 8.59, *R*^2 ^= 0.048, *p* = 0.001, and *F* = 4.35, *R*^2 ^= 0.024, *p* = 0.001, respectively) and the JSD (*F* = 15.99, *R*^2 ^= 0.085, *p* = 0.001, and *F* = 7.4, *R*^2 ^= 0.039, *p* = 0.001, respectively).

We found a significant association of weaker magnitude, between the participant's age, sex, and BMIZ score, and bacterial composition, in some β-diversity measurements. Age was significantly associated with the Bray-Curtis dissimilarity index (*F* = 2.13, *R*^2 ^= 0.012, *p* = 0.014), the weighted UniFrac (*F* = 2.82, *R*^2^ = 0.016, *p* = 0.003) and the JSD (*F* = 2.64, *R*^2 ^= 0.014, *p* = 0.011). BMIZ was significantly associated with bacterial composition when measured by the Bray-Curtis dissimilarity method (*F* = 1.72, *R*^2 ^= 0.010, *p* = 0.03) and the JSD (*F* = 1.94, *R*^2 ^= 0.010, *p* = 0.046), while sex was significantly associated the weighted UniFrac index (*F* = 1.88, *R*^2 ^= 0.011, *p* = 0.043).

The multivariate model using the JSD method explained the overall highest amount of variance 16.6% of the variation in bacterial composition (Figure 2D). The remaining models explained a smaller amount of β-diversity variation: 12.8%, 11.8% and 8.3% for the Bray-Curtis dissimilarity, the weighted UniFrac and the unweighted UniFrac, respectively.

### 3.4. Taxonomic alterations associated with FSIQ score

**Figure 3:**
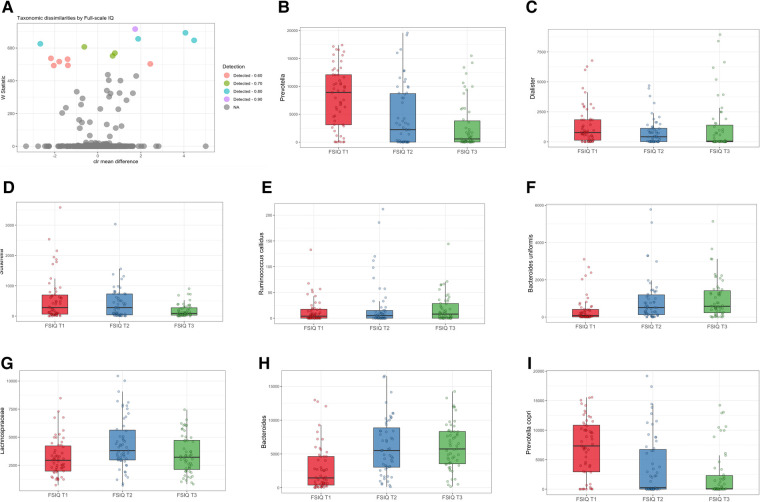
Differentially abundant taxa associated with full-scale IQ scores. (**A**) Volcano plot showing differentially abundant s-OTUs associated with FSIQ scores in the whole cohort, as detected by ANCOM. The *x*-axis represents the difference in mean centered log ratio (clr)-transformed abundance between groups, and the *y*-axis represents the ANCOM W Statistic. s-OTU points are colored by the level of ANCOM significance, with 0.9 being the highest level; s-OTUs in gray were not significant. (**B**–**I)** Boxplots of clr-transformed abundance of s-OTUs significantly associated with FSIQ scores, adjusted for sex, age, SES score, hemoglobin level, HAZ and BMIZ scores. Tertiles of FSIQ were categorized as low, middle and high FSIQ score tertiles, T1 being the lowest tertile (FSIQ scores between 59 and 96), T2 the middle tertile (FSIQ scores between 97 and105), and T3 the highest tertile (FSIQ scores between 106 and 127). The midline in the box plots represents the median, the lower bound of the box represents the 25th percentile, the upper bound of the box represents the 75th percentile, the lowest point of the lower whisker represents the minimum and the highest point of the upper whisker represents the maximum. FSIQ, full-scale IQ; s-OTUs, sub-operational taxonomic units; ANCOM, analysis of composition of microbiomes; SES, socioeconomic status; HAZ, height for age *z*-score at infancy (18–30 months); BMIZ, body mass index *z*-score at age 6–9 years.

In agreement with the profound differences of bacterial composition, we found significant associations between the relative abundance of several bacterial genera, with adjustment for age, sex, SES score, hemoglobin, HAZ and BMIZ scores (Figure 3A). Genus *Prevotella* was detected at the highest detection level (*W*_stat _= 706), followed by *Dialister* (*W*_stat _= 675), *Sutterella* (*W*_stat _= 637), *Ruminococcus callidus* (*W*_stat _= 609), *Bacteroides uniformis* (*W*_stat _= 605) and Lachnospiraceae (*W*_stat _= 553). At a lower detection level, there was an association with *Bacteroides*, *Prevotella copri*, *Oscillospira* and *Clostridium* (Supplementary Table S3). FSIQ scores were inversely associated with the relative abundance of *Prevotella* (including species *Prevotella copri*), *Dialister* and *Sutterella*, while *Ruminococcus callidus, Bacteroides uniformis and* Lachnospiraceae were characterized by a positive association with FSIQ levels (Figures 3B–I).

An unadjusted analysis revealed significant correlations between FSIQ scores and *Prevotella* (Spearman's *r* = −0.42, *p* < 0.0001), *Dialisted* (Spearman's *r* = −0.19, *p* = 0.012), *Sutterella* (Spearman's *r* = −0.2, *p* = 0.01), the species *Ruminococcus callidus* (Spearman's *r* = 0.27, *p* = 0.001), and *Bacteroides uniformis* (Spearman's *r* = 0.41, *p* < 0.0001; Supplementary Figures S7A–E).

**Figure 4:**
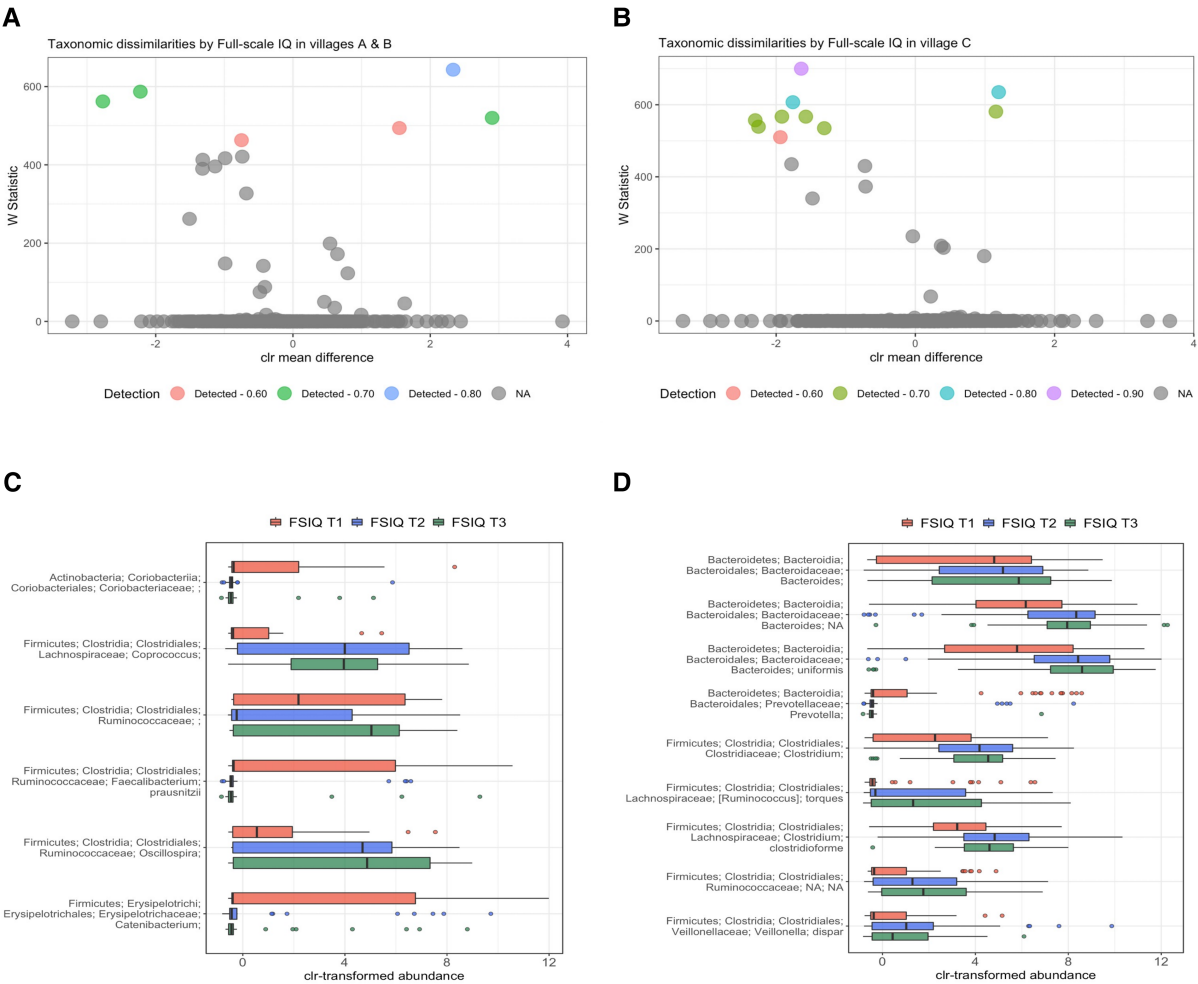
Differentially abundant taxa by village of residence and socioeconomic status. (**A,B**) Volcano plots showing differentially abundant s-OTUs as detected by ANCOM, stratified by village; A/B [high/intermediate SES] (**A**), and C [low SES] (**B**). The *x*-axis represents the difference in mean centered log ratio (clr)-transformed abundance between groups, and the *y*-axis represents the ANCOM W Statistic. s-OTU points are colored by level of ANCOM significance, with 0.9 being the highest level; s-OTUs in gray were not significant. **(B,C)** Boxplots of clr-transformed abundance of s-OTUs significantly associated with FSIQ scores in villages A/B [high/intermediate SES] (**C**) and in village C [low SES] (**D**), adjusted for sex, age, SES score, hemoglobin level (g/dl), HAZ and BMIZ scores. Tertiles of FSIQ were categorized as low, middle and high FSIQ score tertiles, T1 being the lowest tertile (FSIQ scores between 59 and 96), T2 the middle tertile (FSIQ score between 97 and 105), and T3 the highest tertile (FSIQ scores between 106 and 127). The mid line in the box plots represents the median, the lower bound of the box represents the 25th percentile, the upper bound of the box represents the 75th percentile, the lowest point of the lower whisker represents the minimum and the highest point of the upper whisker represents the maximum. FSIQ, full-scale IQ; s-OTUs, sub-operational taxonomic units; ANCOM, analysis of composition of microbiomes; SES- socioeconomic status; HAZ, height for age *z*-score at infancy (18–30 months); BMIZ, body mass index *z*-score at age 6–9 years.

Since a significant percentage of bacterial variance was explained by SES scores, we performed a stratified analysis by village of residence. We found a consistent bacterial variation associated with FSIQ score in all villages, thus independent from SES and adjusted for the aforementioned covariates (Figures 4A,B). Among children from village C (low SES), *Bacteroides uniformis* was the most strongly associated species with FSIQ score (*W*_stat _= 700), followed by *Prevotella* (*W*_stat _= 635), the species *Clostridioforme* (*W*_stat _= 607), including the lower taxonomic levels Clostridiales (*W*_stat _= 581) and *Clostridium* (*W*_stat _= 567), *Veillonella dispar* (*W*_stat _= 567), *Bacteroides* (*W*_stat _= 557) and *Ruminococcus torques* (*W*_stat _= 539). Importantly, FSIQ score was positively associated with the relative abundance of Bacteroides, including *Bacteroides uniformis*, Clostridium, including species *Clostridioforme*, Ruminococcaceae including *Ruminococcus torques* and *Veillonella dispar*, while Prevotella was inversely associated with FSIQ score (Figure 4C).

FSIQ score of children from the higher SES villages (A/B), was associated with altered relative abundance of *Faecalibacterium prausnitzii* (*W*_stat _= 643), *Oscillospira* (*W*_stat _= 587), *Coprococcus* (*W*_stat _= 562) and *Catenibacterium* (*W*_stat _= 520). The full results of the stratified ANCOM analysis are presented in Supplementary Table S4. Notably, *Coprococcus*, *Ruminococcaceae*, including genus *Oscillospira* were positively associated with increased FSIQ score, while *Coriobacteriaceae*, *Faecalibacterium prausnitzii* and *Catenibacterium* levels were depleted with increasing FSIQ score (Figure 4D).

## 4. Discussion

We characterized the cognitive development of school-age children, in association with intestinal microbiome diversity and composition and environmental exposures, including SES, a major factor that influences both cognitive development and the gut microbiome.

We found a significant association between microbial α-diversity, measured by both Shannon's diversity and Pielou's evenness indices and FSIQ scores. There was a progressive increase in both diversity indices with increased FSIQ score. The Shannon diversity index accounts for both richness and evenness of s-OTUs and largely mirrors the evenness findings of this cohort. In general, lower values of Shannon diversity index indicate less diversity, thus the intestinal microbiome of children with lower FSIQ scores was less diverse, less even, and less rich compared to children with higher scores. Correspondingly, Pielou's evenness considers the number of species and the relative abundance of species in a sample. We found a positive relationship between evenness and FSIQ score. Lower values of Pielou's index represent less even distributions of species, thus implying potential dominance of some species in the gut. Therefore, in our study, lower FSIQ scores were associated with less evenness of species inhabiting the human gut. We also found significant differences in the intestinal microbiome composition according to FSIQ score. These associations were observed even after adjustment for SES and nutritional status, measured by hemoglobin levels, BMIZ and HAZ scores.

A limited number of studies examined the associations of the gut microbiome diversity and composition with children's developments, mainly between ages 2 of 3 years, (20, 54, 55). These ages are usually characterized by profound changes in the gut microbiome until it stabilizes. Unlike our study, Carlson et al. (20) in their study of 89 children aged 1–2 years demonstrated that greater α-diversity was associated with poorer cognitive performance. Streit et al. studied 323 children aged 45 months and showed weak negative correlations between alpha diversity, as measured by Faith phylogenetic diversity index and FSIQ (correlation coefficient ranged between −0.10 and −0.14), but such differences were not observed for other indices of alpha diversity when adjusting for confounders and multiple comparisons (54). Rorthenberg and colleagues studied 46 children from rural China, and reported no significant association between alpha diversity and cognitive development at age 3 years (55). The negative associations between alpha diversity and cognitive development in prior studies (20, 54), might be unexpected, since higher α-diversity usually indicates a more mature, adult-like community, while reduced α-diversity is commonly associated with poor health outcomes, including metabolic and inflammatory bowel diseases (1, 56). The differences between our finding of a positive relationship between alpha diversity and others (20, 54) showing inverse associations might be related to discrepancies in the study population, specifically, our study included school-age children that likely have adult-like stable microbiomes, while other studies mainly included infants and pre-school children, characterized by a microbiome that is still evolving.

Our model was adjusted for SES and we observed a strong and significant association between SES and cognitive performance. These results are in line with existing evidence, demonstrating the profound influence of SES on cognitive development, and behavioral outcomes (57), and on the gut microbiome at school age (25). This complex interplay between environmental exposures, the intestinal microbiome, and individual neurodevelopment emphasizes the need for tailored developmental programs and policies that are designed to alleviate SES-related disparities in cognitive performance in children.

We found significant associations between the intestinal microbiome composition and FSIQ score, which were consistent in four distance measures for quantifying β-diversity: the binary Bray-Curtis, the JSD, and the phylogenetic weighted and unweighted UniFrac. Cognitive performance explained the highest percentage of variance in all methods, followed by the SES score. Age was significantly, yet more finely associated with microbial composition. Overall, JSD was the most sensitive method capturing 16.6% of the variance. While the FSIQ score explained the most variance, SES score was an important determinant of β-diversity, suggesting that these factors independently influence both the intestinal microbiome and cognitive performance. The composition of the gut microbiome is a complex trait, with the quantitative variation in the microbiome affected by a large number of host and environmental factors, each of which may have only a small additive effect, making it difficult to identify the association for each separate item (58, 59). Falony et al. reported significant relationships between previously unidentified factors such as red blood cell count and hemoglobin levels and fecal bacterial composition (58), while in our study the association between hemoglobin level and beta diversity was not statistically significant, possibly due to the smaller sample size. Our model explained 16% of the variance in microbial composition, a relatively high percentage, nonetheless indicating the need to explore additional contributions for example from dietary habits, stochastic effects, and/or biotic interactions.

While alpha and beta diversity capture complex variation across the community, we observed significant differences in the relative abundance of specific genera and species in association with FSIQ score. High relative abundance of the genera *Prevotella, Dialister* and *Sutterella* was associated with the lower cognitive performance tertiles, while Bacteroides were positively correlated with elevated FSIQ score. Interestingly, Carlson et al. (20) described a strong association between high levels of *Bacteroides* with the highest level of cognitive performance at 2 years old. Similarly, Tamana et al. (60) showed that *Bacteroidetes*-dominant gut microbiome and higher relative abundance of genus *Bacteroides* in late infancy were associated with enhanced neurodevelopment by the age of 2 years among Canadian children, mainly among males (60). It was also found that *Bacteroidetes*-dominant microbiome was enriched with numerous metabolic functions including sphingolipid metabolism and glycosphingolipid biosynthesis. Furthermore, genes involved in metabolism of folate, biotin, pyruvate, vitamin B6, lipoic acid and fatty acid biosynthesis were enriched in *Bacteroidetes*-dominant microbial composition (60). The intestinal microbiome at 6–9 years is substantially different and more diverse compared to age two years, thus our study adds new knowledge regarding potential involvement of the gut microbiome with cognitive function at school age and not only in early life, when the gut microbiome is less mature, and still affected by early life factors, such as delivery mode (61). Collectively these findings support the idea of microbiota gut-brain-axis during childhood.

Nutrition and dietary patterns are considered major determinants of cognitive performance in children and adolescents (62, 63). For example, iron deficiency anemia is linked to lower neurodevelopmental achievements (28). We measured hemoglobin levels as a proxy for iron deficiency anemia, and included this parameter in all multivariable models, suggesting that the gut microbiome was associated with cognitive function regardless of hemoglobin levels. Additional nutritional factors might influence cognitive performance and academic achievements, including B vitamins, e.g., folate, vitamin B12 (64–66), and overall dietary patterns (63). Diet in turn affects the development of the intestinal microbiome in early life (67). We also found altered composition of the gut microbiome in relation to dietary intake of polyunsaturated fatty acids in a different cohort of Arab children aged 10–12 years (68). Our study lacks information on dietary intake of this cohort. Therefore, the associations between intestinal microbiome and cognitive function in children should be further explored in large-scale prospective studies, while deciphering potential mechanistic role of dietary intake and nutritional status in such associations and possibly intervention studies including probiotic or prebiotic supplementation to assess the association with the microbiome and cognitive performance.

Our study has some limitations. First, this is a cross-sectional study design, thus the directionality of the observed association between the gut microbiome and cognitive development remains to be determined in prospective studies. Second, diet is one of the main determinants of the gut microbiome and affects the (development) of cognitive (dys)function (24), and we did not obtain detailed dietary questionnaires for the participants. In the current cohort, early life dietary exposures were relatively similar and characterized by a high prevalence of breastfeeding, a similar age of first exposure to solid foods, and a late entrance to a daycare. Moreover, since all participants belong to the same ethnic group, they share common dietary practices, mainly a high prevalence of home-cooked, traditional meals and a diet rich in fruit, vegetables, and legumes. Nevertheless, at school-age, there are uncontrolled dietary exposures, that might have an important association with the child's microbiome and cognitive function.

We used archived stool samples that were collected during 2007–2009. There might have been changes over time, that might affect both cognitive development and the gut microbiome. The SES rank of the study villages remained stable during over one decade (69). Conversely, changes in diet and nutritional status were documented (70, 71), which might influence both the gut microbiome and cognitive development. Although, these changes should not affect the observed association between the gut microbiome and cognitive development in our study, future studies using specimens reflecting an up-to-date host-microbiome-environment interaction are needed.

The strengths of the current study include a relatively large cohort of healthy school-age children, with a defined geographic, ethnic, and cultural background, yet divergent SES. Geographic residency and ethnicity are strong modulators of the intestinal microbiome (72), thus the associations demonstrated in the current study are independent from these important confounders. Moreover, our results were adjusted for potential confounders that affect both the microbiome and FSIQ score.

## 5. Conclusions

We demonstrated significant associations between the gut microbiome and cognitive development in healthy school age children, independent of SES. Future longitudinal studies are needed to understand the directionality of the associations and mechanisms that might explain these relationships.

## Data Availability

Data generated in the framework of the study cannot be made publicly available due to legal and ethical restrictions. Aggregate anonymized data might be available upon request from the corresponding author.

## References

[1] Clemente JC Ursell LK Parfrey LW Knight R. The impact of the gut microbiota on human health: an integrative view. Cell. (2012) 148(6):1258–70. 10.1016/j.cell.2012.01.035 22424233 PMC5050011

[2] McDonald D Hyde E Debelius JW Morton JT Gonzalez A Ackermann G American gut: an open platform for citizen science microbiome research. mSystems. (2018) 3(3):e00031–18. 10.1128/mSystems.00031-18 PMC5954204 29795809

[3] Vuong HE Yano JM Fung TC Hsiao EY. The microbiome and host behavior. Annu Rev Neurosci. (2017) 40:21–49. 10.1146/annurev-neuro-072116-031347 28301775 PMC6661159

[4] Carabotti M Scirocco A Maselli MA Severi C. The gut-brain axis: interactions between enteric microbiota, central and enteric nervous systems. Ann Gastroenterol. (2015) 28(2):203–9. PMID: ; PMCID: 25830558 PMC4367209

[5] Provensi G Schmidt SD Boehme M Bastiaanssen TFS Rani B Costa A Preventing adolescent stress-induced cognitive and microbiome changes by diet. Proc Natl Acad Sci U S A. (2019) 116(19):9644–51. 10.1073/pnas.1820832116 31010921 PMC6511019

[6] Heyck M Ibarra A. Microbiota and memory: a symbiotic therapy to counter cognitive decline? Brain Circ. (2019) 5(3):124–9. 10.4103/bc.bc_34_19 31620659 PMC6785944

[7] Foster JA Rinaman L Cryan JF. Stress & the gut-brain axis: regulation by the microbiome. Neurobiol Stress. (2017) 7:124–36. 10.1016/j.ynstr.2017.03.001 29276734 PMC5736941

[8] Arnoriaga-Rodriguez M Fernandez-Real JM. Microbiota impacts on chronic inflammation and metabolic syndrome—related cognitive dysfunction. Rev Endocr Metab Disord. (2019) 20(4):473–80. 10.1007/s11154-019-09537-5 31884557

[9] Vuong HE Hsiao EY. Emerging roles for the gut microbiome in autism spectrum disorder. Biol Psychiatry. (2017) 81(5):411–23. 10.1016/j.biopsych.2016.08.024 27773355 PMC5285286

[10] Dinan TG Cryan JF. Gut instincts: microbiota as a key regulator of brain development, ageing and neurodegeneration. J Physiol. (2017) 595(2):489–503. 10.1113/JP273106 27641441 PMC5233671

[11] Liu F Li J Wu F, Zheng H Peng Q Zhou H. Altered composition and function of intestinal microbiota in autism spectrum disorders: a systematic review. Transl Psychiatry. (2019) 9(1):43. 10.1038/s41398-019-0389-6 30696816 PMC6351640

[12] Gao W Salzwedel AP Carlson AL Xia K Azcarate-Peril MA Styner MA Gut microbiome and brain functional connectivity in infants-a preliminary study focusing on the amygdala. Psychopharmacology (Berl). (2019) 236(5):1641–51. 10.1007/s00213-018-5161-8 30604186 PMC6599471

[13] O'Mahony SM Clarke G Dinan TG Cryan JF. Early-life adversity and brain development: is the microbiome a missing piece of the puzzle? Neuroscience. (2017) 342:37–54. 10.1016/j.neuroscience.2015.09.068 26432952

[14] Gilmore JH Knickmeyer RC Gao W. Imaging structural and functional brain development in early childhood. Nat Rev Neurosci. (2018) 19(3):123–37. 10.1038/nrn.2018.1 29449712 PMC5987539

[15] Jena A Montoya CA Mullaney JA Dilger RN Young W McNabb WC, Gut-brain axis in the early postnatal years of life: a developmental perspective. Front Integr Neurosci. (2020) 14:44. 10.3389/fnint.2020.00044 32848651 PMC7419604

[16] Codagnone MG Stanton C O'Mahony SM Dinan TG Cryan JF. Microbiota and neurodevelopmental trajectories: role of maternal and early-life nutrition. Ann Nutr Metab. (2019) 74(Suppl 2):16–27. 10.1159/000499144 31234188

[17] Luczynski P McVey Neufeld KA Oriach CS Clarke G Dinan TG Cryan JF. Growing up in a bubble: using germ-free animals to assess the influence of the gut microbiota on brain and behavior. Int J Neuropsychopharmacol. (2016) 19(8):pyw020. 10.1093/ijnp/pyw020 26912607 PMC5006193

[18] Christian LM Galley JD Hade EM Schoppe-Sullivan S Kamp Dush C Bailey MT. Gut microbiome composition is associated with temperament during early childhood. Brain Behav Immun. (2015) 45:118–27. 10.1016/j.bbi.2014.10.018 25449582 PMC4342262

[19] Wang Y Chen X Yu Y, Liu Y Zhang Q Bai J. Association between gut microbiota and infant’s temperament in the first year of life in a Chinese birth cohort. Microorganisms. (2020) 8(5):753. 10.3390/microorganisms8050753 32429579 PMC7285300

[20] Carlson AL Xia K Azcarate-Peril MA Goldman BD Ahn M Styner MA Infant gut microbiome associated with cognitive development. Biol Psychiatry. (2018) 83(2):148–59. 10.1016/j.biopsych.2017.06.021 28793975 PMC5724966

[21] Koenig JE Spor A Scalfone N Fricker AD Stombaugh J Knight R Succession of microbial consortia in the developing infant gut microbiome. Proc Natl Acad Sci U S A. (2011) 108(Suppl 1):4578–85. 10.1073/pnas.1000081107 20668239 PMC3063592

[22] Derrien M Alvarez AS de Vos WM. The gut microbiota in the first decade of life. Trends Microbiol. (2019) 27(12):997–1010. 10.1016/j.tim.2019.08.001 31474424

[23] John CC Black MM Nelson CA III. Neurodevelopment: the impact of nutrition and inflammation during early to middle childhood in low-resource settings. Pediatrics. (2017) 139(Suppl 1):S59–71. 10.1542/peds.2016-2828H 28562249 PMC5694688

[24] Rothschild D Weissbrod O Barkan E Kurilshikov A Korem T Zeevi D Environment dominates over host genetics in shaping human gut microbiota. Nature. (2018) 555(7695):210–5. 10.1038/nature25973 29489753

[25] Lapidot Y Reshef L Maya M Cohen D Gophna U Muhsen K. Socioeconomic disparities and household crowding in association with the fecal microbiome of school-age children. NPJ Biofilms Microbiomes. (2022) 8(1):10. 10.1038/s41522-022-00271-6 35241676 PMC8894399

[26] Acosta AM de Burga RR Chavez CB Flores JT Olotegui MP Pinedo SR Early childhood cognitive development is affected by interactions among illness, diet, enteropathogens and the home environment: findings from the MAL-ED birth cohort study. BMJ Glob Health. (2018) 3(4):e000752. 10.1136/bmjgh-2018-000752 30058645 PMC6058175

[27] Jefferis BJ Power C Hertzman C. Birth weight, childhood socioeconomic environment, and cognitive development in the 1958 British birth cohort study. Br Med J. (2002) 325(7359):305. 10.1136/bmj.325.7359.305 12169505 PMC117769

[28] Lozoff B Jimenez E Wolf AW. Long-term developmental outcome of infants with iron deficiency. N Engl J Med. (1991) 325(10):687–94. 10.1056/NEJM199109053251004 1870641

[29] Lozoff B Jimenez E Smith JB. Double burden of iron deficiency in infancy and low socioeconomic status: a longitudinal analysis of cognitive test scores to age 19 years. Arch Pediatr Adolesc Med. (2006) 160(11):1108–13. 10.1001/archpedi.160.11.1108 17088512 PMC1866361

[30] Pivina L Semenova Y Doşa MD Dauletyarova M Bjørklund G. Iron deficiency, cognitive functions, and neurobehavioral disorders in children. J Mol Neurosci. (2019) 68(1):1–10. 10.1007/s12031-019-01276-1 30778834

[31] Muleviciene A D'Amico F Turroni S Candela M Jankauskiene A. Iron deficiency anemia-related gut microbiota dysbiosis in infants and young children: a pilot study. Acta Microbiol Immunol Hung. (2018) 65(4):551–64. 10.1556/030.65.2018.045 30418043

[32] Soriano-Lerma A García-Burgos M Alférez MJM Pérez-Carrasco V Sanchez-Martin V Linde-Rodríguez Á Gut microbiome-short-chain fatty acids interplay in the context of iron deficiency anaemia. Eur J Nutr. (2022) 61(1):399–412. 10.1007/s00394-021-02645-6 34383140

[33] Mayneris-Perxachs J Amaral W Lubach GR Lyte M Phillips GJ Posma JM Gut microbial and metabolic profiling reveal the lingering effects of infantile iron deficiency unless treated with iron. Mol Nutr Food Res. (2021) 65(8):e2001018. 10.1002/mnfr.202001018 33599094 PMC8216173

[34] Israel Central Bureau of Statistics. Statistical abstract of Israel 2008. Jerusalem: Israel Central Bureau of Statistics (2009).

[35] Israel Central Bureau of Statistics. Statistical abstract of Israel 2021. Jerusalem, Israel: Israel Central Bureau of Statistics (2021).

[36] Israel Central Bureau of Statistics. Characterization and classification of local authorities by the socio-economic level of the population 2006. Jerusalem, Israel: Israel Central Bureau of Statistics (2009).

[37] Clarfield AM Manor O Nun GB Shvarts S Azzam ZS Afek A Health and health care in Israel: an introduction. Lancet. (2017) 389(10088):2503–13. 10.1016/S0140-6736(17)30636-0 28495109

[38] Muhsen K Barak M Henig C Alpert G Ornoy A Cohen D. Is the association between Helicobacter pylori infection and anemia age dependent? Helicobacter. (2010) 15(5):467–72. 10.1111/j.1523-5378.2010.00793.x 21083753

[39] Muhsen K Ornoy A Akawi A Alpert G Cohen D. An association between Helicobacter pylori infection and cognitive function in children at early school age: a community-based study. BMC Pediatr. (2011) 11:43. 10.1186/1471-2431-11-43 21612616 PMC3121602

[40] Beer-Davidson G Hindiyeh M Muhsen K. Detection of Helicobacter pylori in stool samples of young children using real-time polymerase chain reaction. Helicobacter. (2018) 23(1):e12450. 10.1111/hel.12450 29181860

[41] Naqib A Poggi S Wang W Hyde M Kunstman K Green SJ. Making and sequencing heavily multiplexed, high-throughput 16S ribosomal RNA gene amplicon libraries using a flexible, two-stage PCR protocol. Methods Mol Biol. (2018) 1783:149–69. 10.1007/978-1-4939-7834-2_7 29767361

[42] Walters W Hyde ER Berg-Lyons D Ackermann G Humphrey G Parada A Improved bacterial 16S rRNA gene (V4 and V4-5) and fungal internal transcribed spacer marker gene primers for microbial community surveys. mSystems. (2016) 1(1):e00009–15. 10.1128/mSystems.00009-15 27822518 PMC5069754

[43] Amir A McDonald D Navas-Molina JA Kopylova E Morton JT Zech Xu Z Deblur rapidly resolves single-nucleotide community sequence patterns. mSystems. (2017) 2(2):e00191–16. 10.1128/mSystems.00191-16 28289731 PMC5340863

[44] Bolyen E Rideout JR Dillon MR Bokulich NA Abnet CC Al-Ghalith GA Reproducible, interactive, scalable and extensible microbiome data science using QIIME 2. Nat Biotechnol. (2019) 37(8):852–7. 10.1038/s41587-019-0209-9 31341288 PMC7015180

[45] McDonald D Price MN Goodrich J Nawrocki EP DeSantis TZ Probst A An improved greengenes taxonomy with explicit ranks for ecological and evolutionary analyses of bacteria and archaea. ISME J. (2012) 6(3):610–8. 10.1038/ismej.2011.139 22134646 PMC3280142

[46] Finotello F Mastrorilli E Di Camillo B. Measuring the diversity of the human microbiota with targeted next-generation sequencing. Brief Bioinform. (2018) 19(4):679–92. 10.1093/bib/bbw119 28025179

[47] Oksanen J Blanchet F Friendly M Kindt R Legendre P McGlinn D Vegan: community ecology package (2019). Available at: https://www.mcglinnlab.org/publication/2019-01-01_oksanen_vegan_2019/

[48] Mandal S Van Treuren W White RA Eggesbo M Knight R Peddada SD. Analysis of composition of microbiomes: a novel method for studying microbial composition. Microb Ecol Health Dis. (2015) 26:27663. 10.3402/mehd.v26.27663 26028277 PMC4450248

[49] de la Cuesta-Zuluaga J Kelley ST Chen Y Escobar JS Mueller NT Ley RE Age- and sex-dependent patterns of gut microbial diversity in human adults. mSystems. (2019) 4(4):e00261–19. 10.1128/mSystems.00261-19 31098397 PMC6517691

[50] Bowyer RCE Jackson MA Le Roy CI Ni Lochlainn M Spector TD Dowd JB Socioeconomic status and the gut microbiome: a TwinsUK cohort study. Microorganisms. (2019) 7(1):17. 10.3390/microorganisms7010017 30641975 PMC6351927

[51] Miller GE Engen PA Gillevet PM Shaikh M Sikaroodi M Forsyth CB Lower neighborhood socioeconomic status associated with reduced diversity of the colonic microbiota in healthy adults. PLoS One. (2016) 11(2):e0148952. 10.1371/journal.pone.0148952 26859894 PMC4747579

[52] Valerio G D'Amico O Adinolfi M Munciguerra A D'Amico R Franzese A. Determinants of weight gain in children from 7 to 10 years. Nutr Metab Cardiovasc Dis. (2006) 16(4):272–8. 10.1016/j.numecd.2005.10.008 16679219

[53] Goldberg S Werbeloff N Fruchter E Portuguese S Davidson M Weiser M. IQ and obesity in adolescence: a population-based, cross-sectional study. Pediatr Obes. (2014) 9(6):419–26. 10.1111/j.2047-6310.2013.00203.x 24339055

[54] Streit F Prandovszky E Send T Zillich L Frank J Sabunciyan S Microbiome profiles are associated with cognitive functioning in 45-month-old children. Brain Behav Immun. (2021) 98:151–60. 10.1016/j.bbi.2021.08.001 34371134

[55] Rothenberg SE Chen Q Shen J Nong Y Nong H Trinh EP Neurodevelopment correlates with gut microbiota in a cross-sectional analysis of children at 3 years of age in rural China. Sci Rep. (2021) 11(1):7384. 10.1038/s41598-021-86761-7 33795717 PMC8016964

[56] Integrative HMPRNC. The integrative human microbiome project. Nature. (2019) 569(7758):641–8. 10.1038/s41586-019-1238-8 31142853 PMC6784865

[57] Buckley L Broadley M Cascio CN. Socio-economic status and the developing brain in adolescence: a systematic review. Child Neuropsychol. (2019) 25(7):859–84. 10.1080/09297049.2018.1549209 30466359

[58] Falony G Joossens M Vieira-Silva S Wang J Darzi Y Faust K, Population-level analysis of gut microbiome variation. Science. (2016) 352(6285):560–4. 10.1126/science.aad3503 27126039

[59] Zhernakova A Kurilshikov A Bonder MJ Tigchelaar EF Schirmer M Vatanen T, Population-based metagenomics analysis reveals markers for gut microbiome composition and diversity. Science. (2016) 352(6285):565–9. 10.1126/science.aad3369 27126040 PMC5240844

[60] Tamana SK Tun HM Konya T Chari RS Field CJ Guttman DS Bacteroides-dominant gut microbiome of late infancy is associated with enhanced neurodevelopment. Gut Microbes. (2021) 13(1):1–17. 10.1080/19490976.2021.1930875 34132157 PMC8210878

[61] Mitchell CM Mazzoni C Hogstrom L Bryant A Bergerat A Cher A Delivery mode affects stability of early infant gut microbiota. Cell Rep Med. (2020) 1(9):100156. 10.1016/j.xcrm.2020.100156 33377127 PMC7762768

[62] Anjos T Altmäe S Emmett P Tiemeier H Closa-Monasterolo R Luque V Nutrition and neurodevelopment in children: focus on NUTRIMENTHE project. Eur J Nutr. (2013) 52(8):1825–42. 10.1007/s00394-013-0560-4 23884402

[63] Leventakou V Roumeliotaki T Sarri K Koutra K Kampouri M Kyriklaki A Dietary patterns in early childhood and child cognitive and psychomotor development: the Rhea mother-child cohort study in Crete. Br J Nutr. (2016) 115(8):1431–7. 10.1017/S0007114516000246 26887648

[64] Nilsson TK Yngve A Böttiger AK Hurtig-Wennlöf A Sjöström M. High folate intake is related to better academic achievement in Swedish adolescents. Pediatrics. (2011) 128(2):e358–65. 10.1542/peds.2010-1481 21746721

[65] Kvestad I Hysing M Shrestha M Ulak M Thorne-Lyman AL Henjum S Vitamin B-12 status in infancy is positively associated with development and cognitive functioning 5 y later in Nepalese children. Am J Clin Nutr. (2017) 105(5):1122–31. 10.3945/ajcn.116.144931 28330909

[66] Gewa CA Weiss RE Bwibo NO Whaley S Sigman M Murphy SP Dietary micronutrients are associated with higher cognitive function gains among primary school children in rural Kenya. Br J Nutr. (2009) 101(9):1378–87. 10.1017/S0007114508066804 18826659

[67] Vandenplas Y Carnielli VP Ksiazyk J Luna MS Migacheva N Mosselmans JM Factors affecting early-life intestinal microbiota development. Nutrition. (2020) 78:110812. 10.1016/j.nut.2020.110812 32464473

[68] Lapidot Y Reshef L Goldsmith R Na'amnih W Kassem E Ornoy A, The associations between diet and socioeconomic disparities and the intestinal microbiome in preadolescence. Nutrients. (2021) 13(8):2645. 10.3390/nu13082645 34444813 PMC8398108

[69] Statistics ICBo. Characterization and classification of geographical units by the socio-economic level of the population, 2017. Jerusalem, Israel: Israel Central Bureau of Statistics (2021).

[70] Peng W Goldsmith R Shimony T Berry EM Sinai T. Trends in the adherence to the Mediterranean diet in Israeli adolescents: results from two national health and nutrition surveys, 2003 and 2016. Eur J Nutr. (2021) 60(7):3625–38. 10.1007/s00394-021-02522-2 33683418

[71] Loewenberg Weisband Y Kaufman-Shriqui V Wolff Sagy Y Krieger M Abu Ahmad W Manor O. Area-level socioeconomic disparity trends in nutritional status among 5–6-year-old children in Israel. Arch Dis Child. (2020) 105(11):1049–54. 10.1136/archdischild-2019-318595 32376694

[72] Gaulke CA Sharpton TJ. The influence of ethnicity and geography on human gut microbiome composition. Nat Med. (2018) 24(10):1495–6. 10.1038/s41591-018-0210-8 30275567

