# Supplementary material for: Relationships of the gut microbiome with cognitive development among healthy school-age children
Source: Front Pediatr. 2023 May 19;11:1198792. doi: 10.3389/fped.2023.1198792 (PMC10235814; doi:10.3389/fped.2023.1198792)
Supplement: Supplementary file 1 [file Datasheet1.pdf]

Relationships of the gut microbiome with cognitive development among healthy school-age children

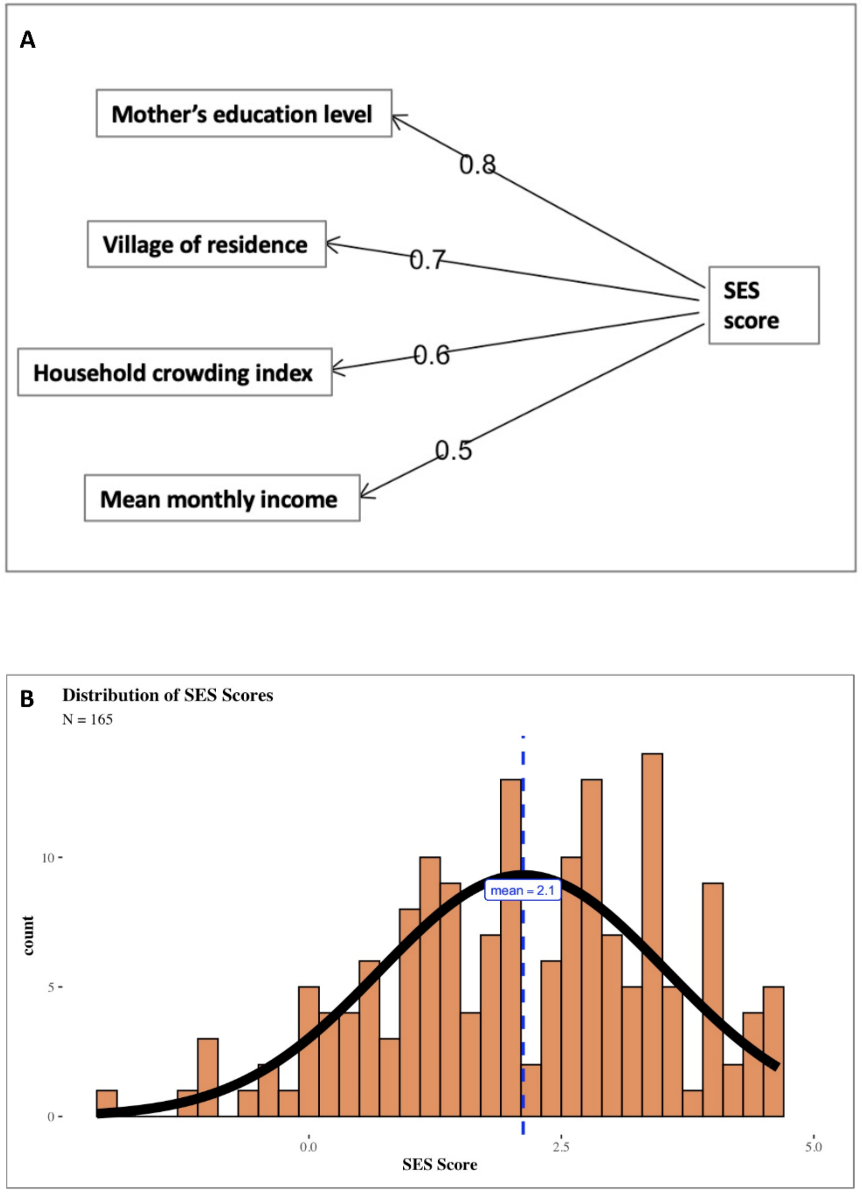

Supplementary Figure 1: (A) The standardized loadings of the selected variables for confirmatory factor analysis for the newly generated socioeconomic status (SES) score. **(B)** A histogram of the distribution SES composite score in the study cohort.

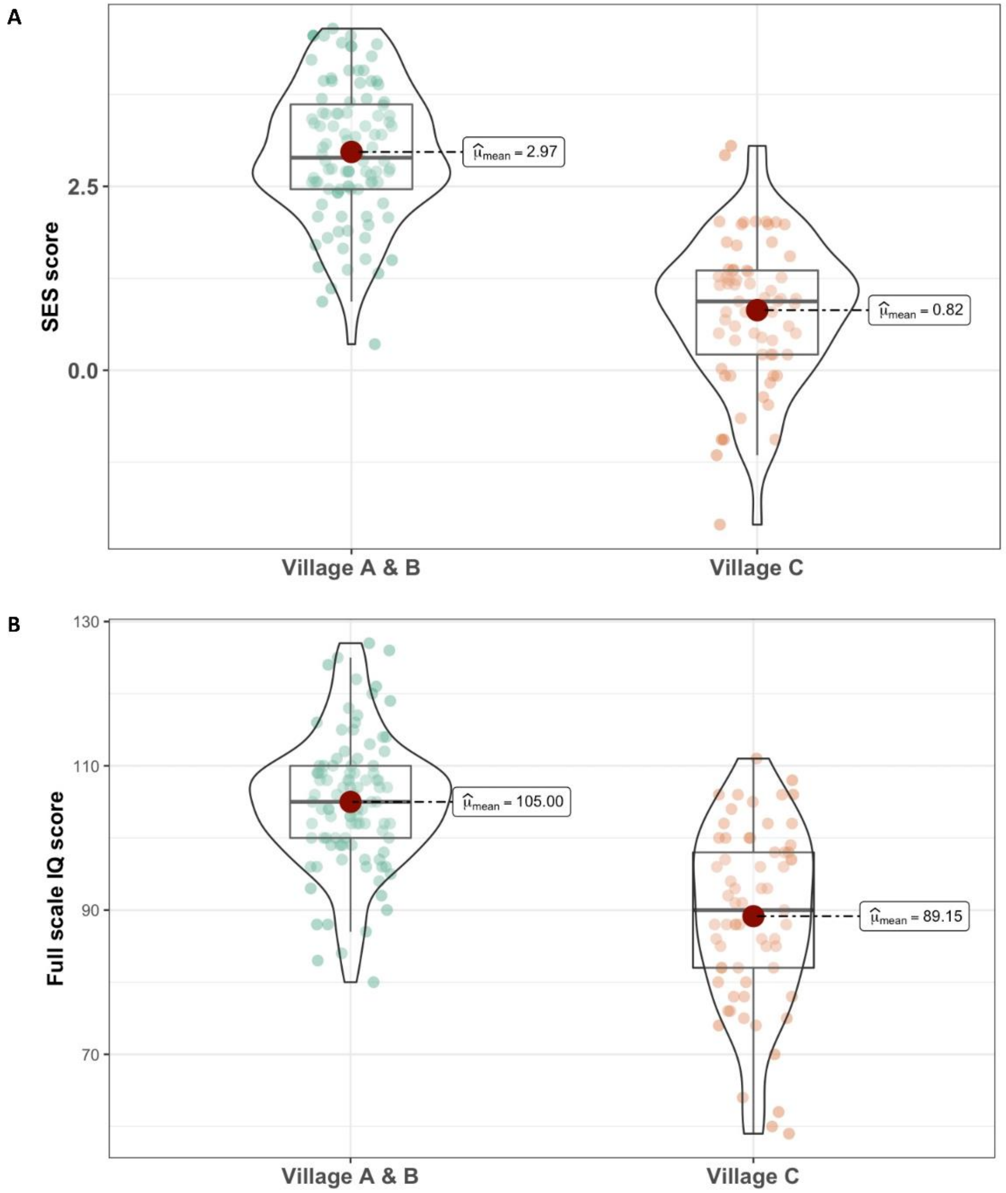

Supplementary Figure 2: (A) Box-violin plots of the composite socioeconomic status (SES) scores across the villages. The mean SES score was significantly higher in villages A/B [high/intermediate SES] compared to village C [low SES] ($p<0.0001$). **(B)** Box-violin plots of Full-scale IQ (FSIQ) scores across the villages. The mean FSIQ score was significantly higher in villages A/B [high/intermediate SES] compared to village C [low SES] ($p<0.0001$).

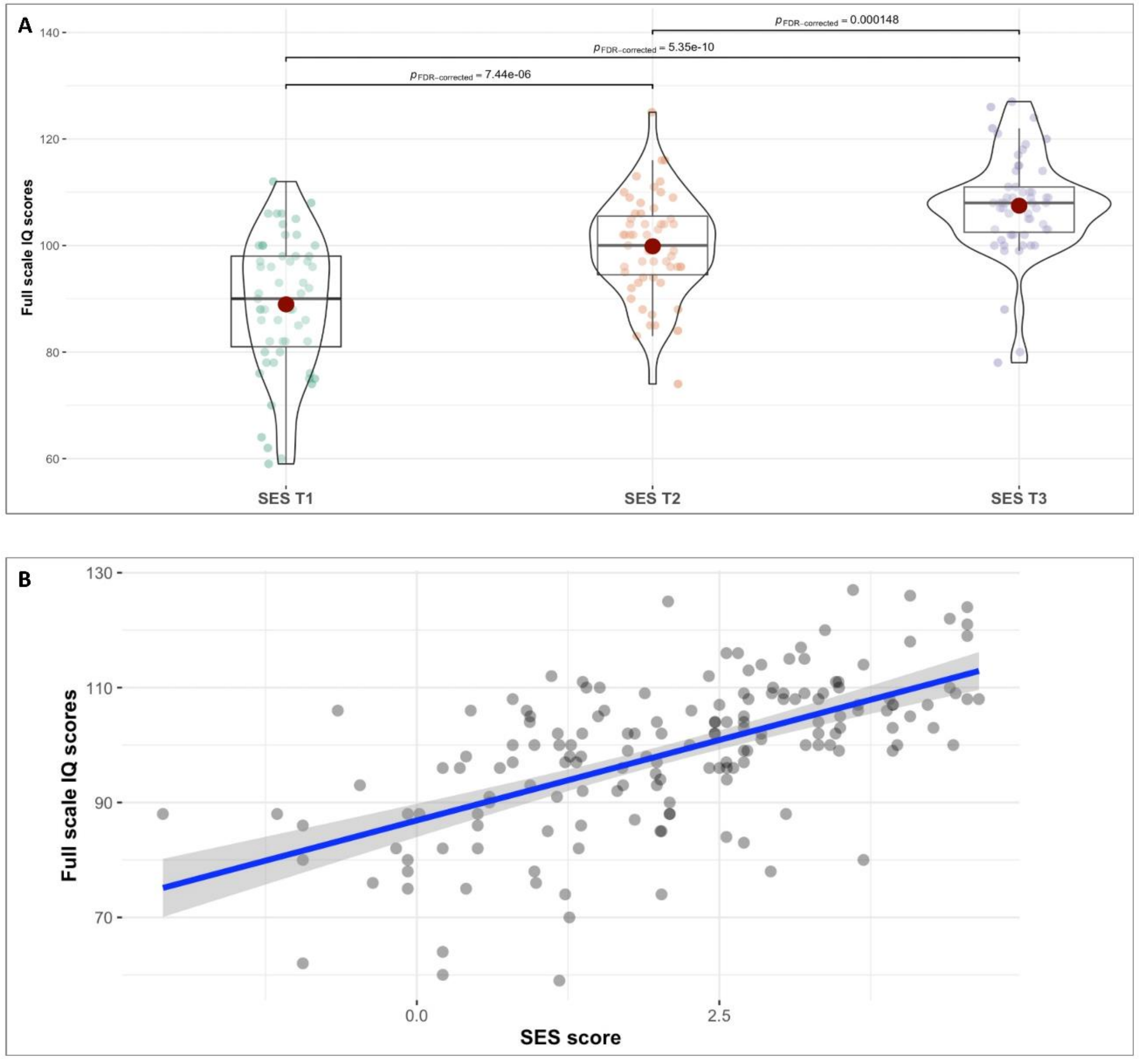

Supplementary Figure 3: (A) Box-violin plots of the Full-scale IQ (FSIQ) scores across tertiles of the composite SES scores (T1 representing the lowest tertile and T3 – the highest tertile). There was a significant increase in FSIQ score with increased SES-score; T1 compared to T2 and T3 ($p<0.001$ for both comparisons) and T2 compared to T3 ($p=0.0001$). Pairwise comparisons were analyzed using Games-Howell test, and FDR correction. **(B)** The strong correlation between participant's socioeconomic status and FSIQ) scores; Pearson's $r= 0.61$, $p< 0.001$.

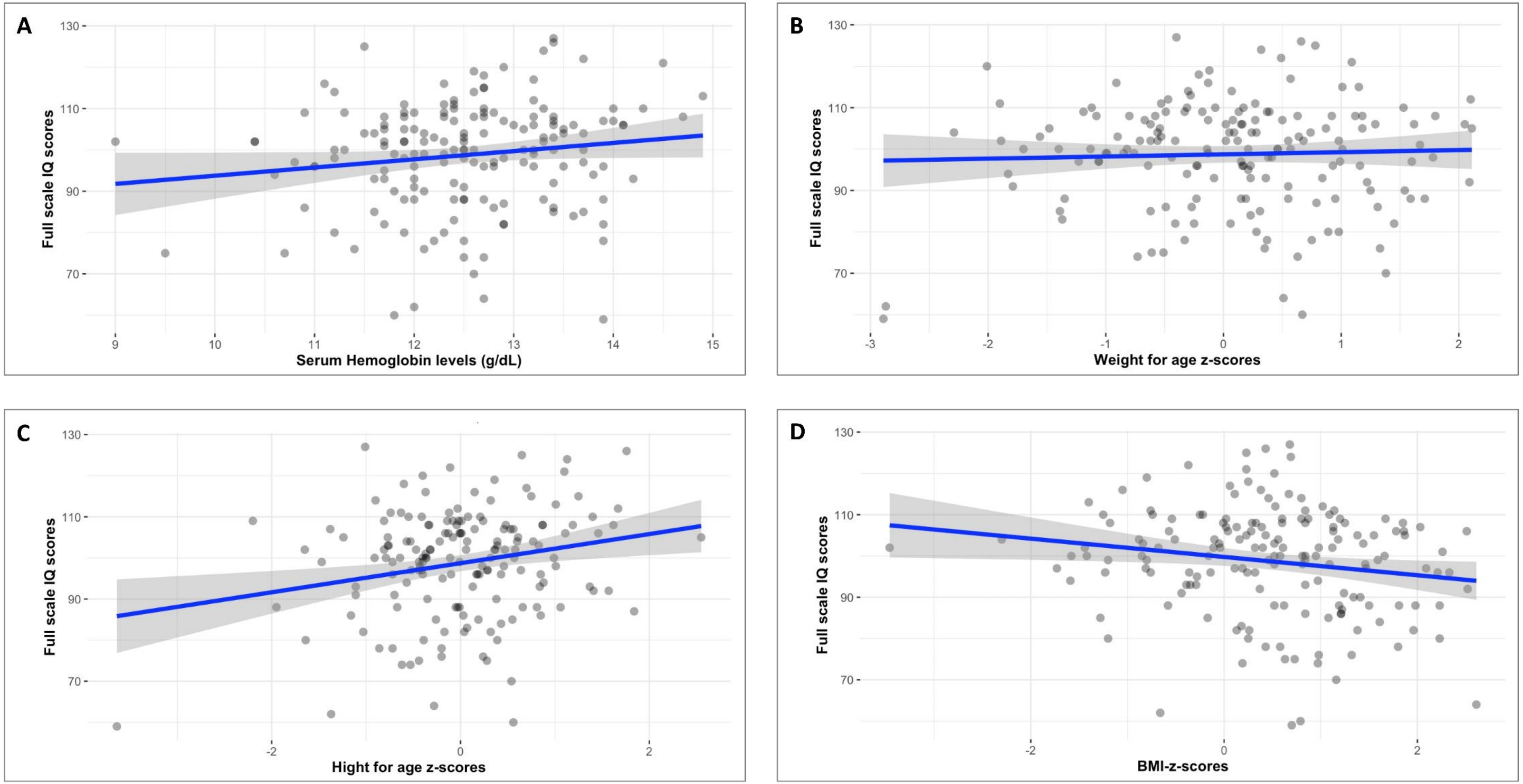

Supplementary Figure 4: (A) The correlation between hemoglobin levels (g/dL) and Full-scale IQ (FSIQ) scores; Pearson's r=0.15, p=0.059.
(B) The correlation between the participant's weight for age z-scores at infancy (18-30 months) and FSIQ scores; Pearson's r=0.04, p=0.617.
(C) The relationship between participant's height for age z-scores at infancy (18-30 months) and FSIQ scores; Pearson's r= 0.22, p=0.004.
(D) The relationship between the participant's BMIZ scores in childhood (6-9 years) and FSIQ scores; Pearson's r= - 0.17, p=0.025.

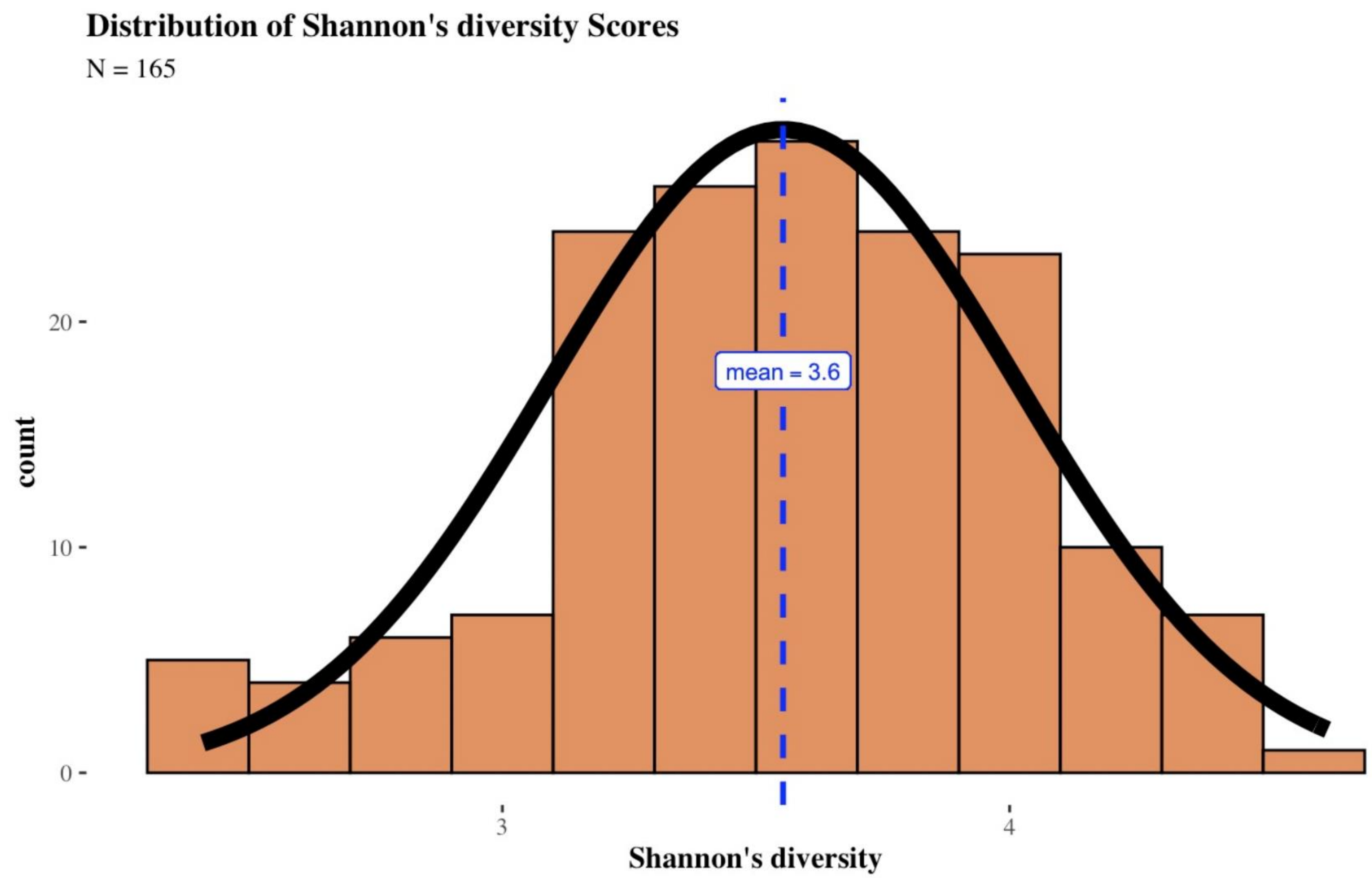

Supplementary figure 5: A histogram displaying the distribution of individuals’ Shannon’s diversity index

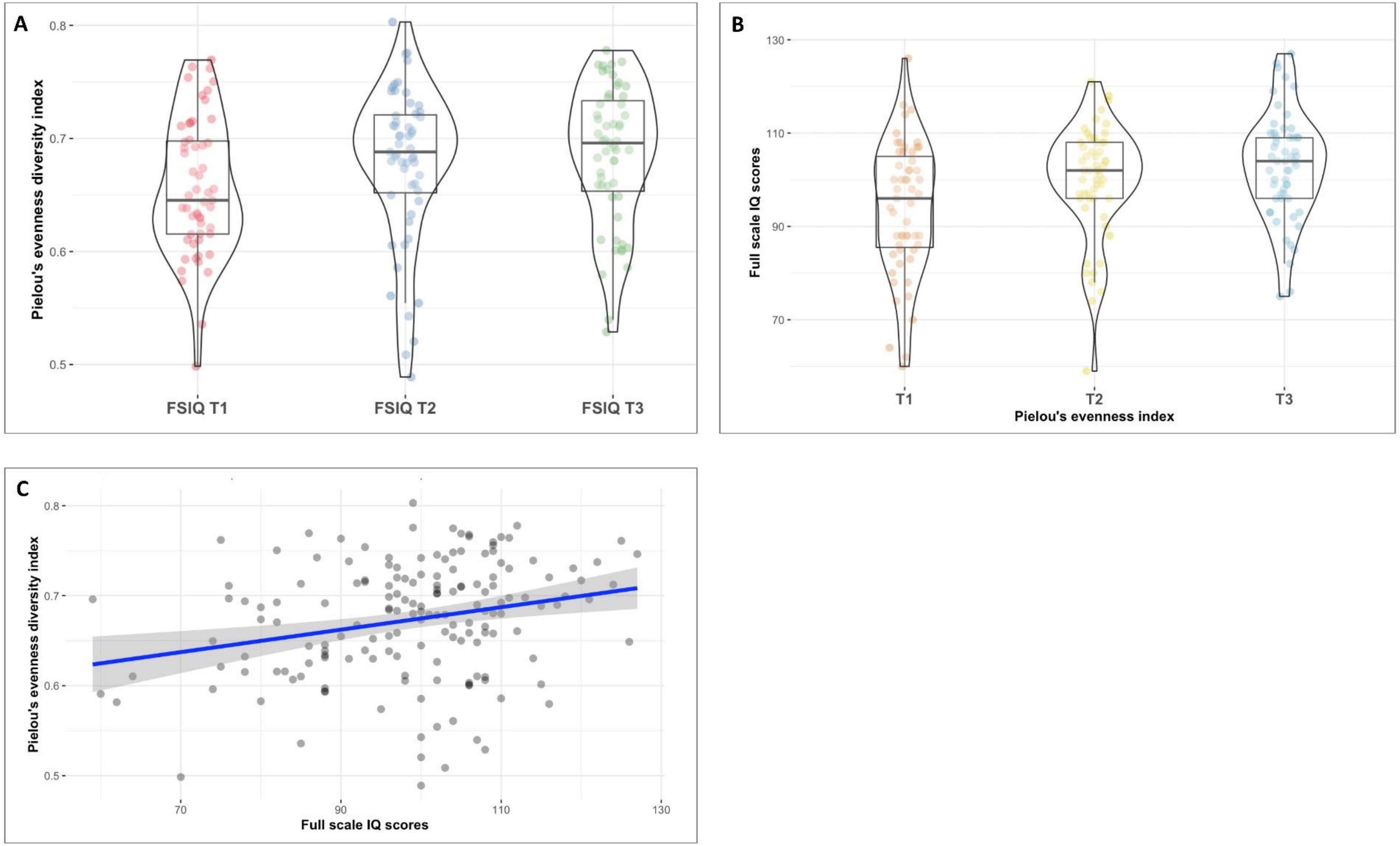

Supplementary figure 6: (**A**) Box-violin plots of microbial diversity, measured by the Pielou's evenness index, across tertiles of Full-scale IQ (FSIQ) score, showing a significant alteration of species evenness with increased FSIQ ($p=0.0009$). (**B**) Box-violin plots of FSIQ score across tertiles of the Pielou's evenness index, showing a significant increase in FSIQ score with alteration of species evenness ($p<0.001$). (**C**) The correlation between Pielou's evenness index and FSIQ score; Spearman's $r = 0.24$, $p=0.002$.

* The x-axis in figures A and C represents tertiles of the presented variable, T1 being the lowest tertile and T3 the highest tertile. ** The midline in the box plots (figures A, B) represents the median, the lower bound of the box represents the 25th percentile, the upper bound of the box represents the 75th percentile, the lowest point of the lower whisker represents the minimum and the highest point of the upper whisker represents the maximum. The violin plot implements a rotated kernel density plot on each side, adding information regarding the full distribution of the measured data; the width of the violin indicates the frequency.

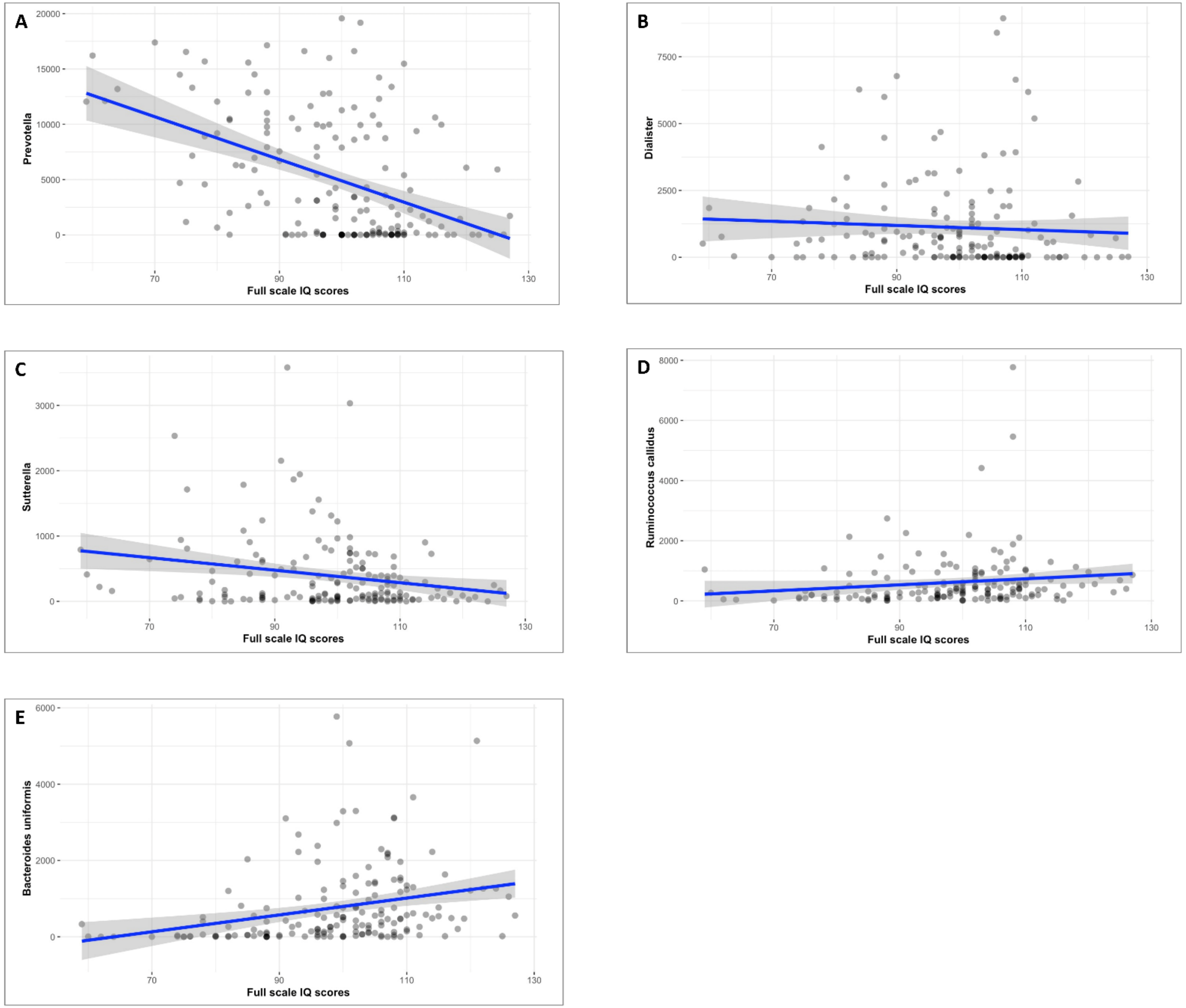

Supplementary figure 7: The correlation between clr-transformed abundance of genera and species associated with FSIQ scores (ANCOM detection level > 0.7); **(A)***Prevotella* (Spearman's r = -0.42, p<0.001), **(B)** Dialister (Spearman's r = -0.19, p=0.012), (**C**) *Sutterella* (Spearman's r = -0.2, p=0.01), (**D**) *Ruminococcus callidus* Spearman's r = 0.27, p=0.001), and (**E**) *Bacteroides uniformis* (Spearman's r = 0.41, p<0.001).
